# Laboratory Findings of Benign Convulsions With Mild Gastroenteritis: A Meta-Analysis

**DOI:** 10.7759/cureus.36784

**Published:** 2023-03-28

**Authors:** Yoshifumi Miyagi, Tomoyuki Sasano, Kentoku Kin

**Affiliations:** 1 Department of Pediatrics, Haibara General Hospital, Shizuoka, JPN; 2 Department of Obstetrics and Gynecology, Osaka Saiseikai Nakatsu Hospital, Osaka, JPN

**Keywords:** chatgpt, uric acid, seizure, meta-analysis, laboratory finding, hyperuricemia, gastroenteritis, convulsion, child, benign convulsions with mild gastroenteritis

## Abstract

Investigating factors associated with benign convulsions with mild gastroenteritis (CwG) is important for early detection and treatment. In previous studies, uric acid (UA) has been reported to be associated with CwG. However, the association between CwG and abnormal laboratory values remains inconclusive. We performed a meta-analysis of recent reports to determine the association between CwG and laboratory findings, including UA, in patients with acute gastroenteritis without convulsions.

We conducted electronic searches of three databases (PubMed, EMBASE, and Cochrane Library) and one scholarly search engine (Google Scholar (Google, Inc., Mountain View, CA, USA)) up to February 2023 for studies on CwG. Eligible studies were observational studies that assessed patients with CwG, reported laboratory data, and stated the presence or absence of convulsions during illness episodes. Patients were children with mild gastroenteritis, with the exposure group developing convulsions and the control group not. The outcome was a comparison of laboratory data between the two groups. The effect size was calculated using the standardized mean difference (SMD), and random-effects models were used for the analysis because of high heterogeneity.

In total, 148 articles were included in this study. After the screening, nine studies, including 8,367 patients, were selected for the meta-analysis. The most prevalent laboratory finding was an increased serum UA level, with an SMD of 1.42 (N = 6,411; 95% confidence interval (CI): (1.12, 1.72); Z = 9.242, p< 0.001; I ^2 ^= 81.68%, p= 0.002). The optimal serum UA cutoff value was 7.21 mg/dL, with an area under the receiver operating characteristic (ROC) curve (AUC) of 0.827 (95% CI: (0.807, 0.846)).

This meta-analysis suggests that CwG is strongly associated with increased serum UA levels. These results demonstrate that more attention should be paid when interpreting laboratory findings in pediatric patients with acute gastroenteritis.

## Introduction and background

Benign convulsions with mild gastroenteritis (CwGs) were first reported by Morooka [1] in 1982. Komori et al. [2] summarized this in 1995. Although the definition of CwG has not yet been established, the following can be recognized from the concepts reported thus far: (1) benign seizures are usually seen in children from six months to three years of age, (2) normal development is noted before and after the seizure, (3) afebrile seizures are associated with acute gastroenteritis, (4) short-lasting recurrent seizures occur within 24 hours (maximally within a few days), (5) no abnormalities are noted in blood tests, spinal fluid tests, electroencephalograms, or brain imaging findings, and (6) there is good prognosis [3].

Although the prognosis is good, repeated seizures cause panic in parents, and the child often requires hospitalization and acute care [4]. Furthermore, investigating the characteristics of CwG may help improve its assessment and management in pediatric emergency departments. Identifying the predictors of CwG-induced convulsions in infants with gastroenteritis is important in clinical practice.

One characteristic of CwG is the absence of abnormal findings on clinical examination. No blood test result abnormalities have been reported [5-7]. However, a growing body of research has shown that CwG may be associated with increased uric acid (UA) levels or decreased sodium (Na) levels [8-16].

Investigating the factors associated with CwG is important for its early detection and treatment. However, the association between CwG and abnormal laboratory values remains inconclusive. We performed a meta-analysis to determine the association between CwG and laboratory findings in patients with acute gastroenteritis without convulsions and further validate the association between UA and CwG from previous papers.

## Review

Material and methods

Data Sources

Ethical approval was not required as this was a retrospective analysis of previously published data. This study was conducted in accordance with the standard guidelines of the Preferred Reporting Items for Systematic Reviews and Meta-Analyses (PRISMA) [17]. Two authors conducted electronic searches of three electronic databases (PubMed, EMBASE, and Cochrane Library) and one scholarly search engine (Google Scholar (Google, Inc., Mountain View, CA, USA)) for eligible studies published up to February 2023.

Study Selection

Pediatric patients diagnosed with acute gastroenteritis were included in this study. The exposure was CwG. We included participants with acute gastroenteritis without convulsions as study controls. The principal outcome of this analysis was the difference in blood test results between children with gastroenteritis, with or without convulsions.

Data Extraction

Data were extracted independently by two authors. The extracted variables were the author’s name, article title, journal name, publication year, study location, study design, sample size, and mean and standard deviation (SD) of laboratory data for acute gastroenteritis. The following keywords were used in our search strategies: (“convulsions with mild gastroenteritis” or “CwG”) and (“clinical characteristics” or “clinical features” or “laboratory” or “serum”). Potentially eligible articles were assessed using inclusion and exclusion criteria. Studies that met all the following criteria were included: (1) observational studies reporting on patients with CwG, (2) studies reporting laboratory data, and (3) studies reporting the presence or absence of convulsions during an illness episode. Studies that met any one of the following criteria were excluded: (1) conference papers, (2) abstracts, (3) commentaries, (4) letters, and (5) insufficient or inaccurate information. We aimed to identify all relevant studies, regardless of their language or publication status (published or preprinted). Two authors independently assessed the risk of bias in the included studies using the Newcastle-Ottawa Scale (NOS) [18].

Statistical Analysis

The pooled effect estimate was the standardized mean difference (SMD). All statistical analyses and figures were prepared in R language (the “meta” and “metafor” packages) and PyMeta (http://pymeta.com/). Cohen’s classification was used to interpret the magnitude of the effect size of the laboratory findings. SMD > 0.8 was considered large [19]. The heterogeneity of the included studies was assessed using the *I*^2^ statistic, with heterogeneity estimates of 25%, 50%, and 75% representing low, moderate, and high heterogeneity, respectively [20]. The confidence interval (CI) was set at 95%. Statistical significance was set at a p-value of 0.05. In cases where the extracted data units differed, numerical conversions were performed to unify them. Heterogeneity was evaluated using the*I*^2^ statistic. If*I*^2^ > 50%, a random-effects model was used. A standard method for detecting outliers defines a study as an outlier if its CI does not overlap with the pooled effect. Sensitivity analysis was performed to assess the possible causes of heterogeneity. All analyses were performed using R version 4.2.2.

Meta-Regression

Meta-regression was used to evaluate the potential sources of heterogeneity among the included studies. Univariate meta-regression analysis was performed to observe the effect of many factors, including age, sex, country, publication year, white blood cell (WBC) count, and Na and calcium (Ca) levels, on the final result.

Subgroup Analysis

We also conducted subgroup analyses to evaluate the etiology of the heterogeneity. Subgroup analyses were performed according to country.

Publication Bias

To assess reporting bias, if at least 10 trials were available for a single intervention, we used funnel plots to examine the reporting bias for the primary outcome. However, the limited number of trials for each intervention precluded the use of this test.

Result

Literature Search and Study Selection

**Figure 1:**
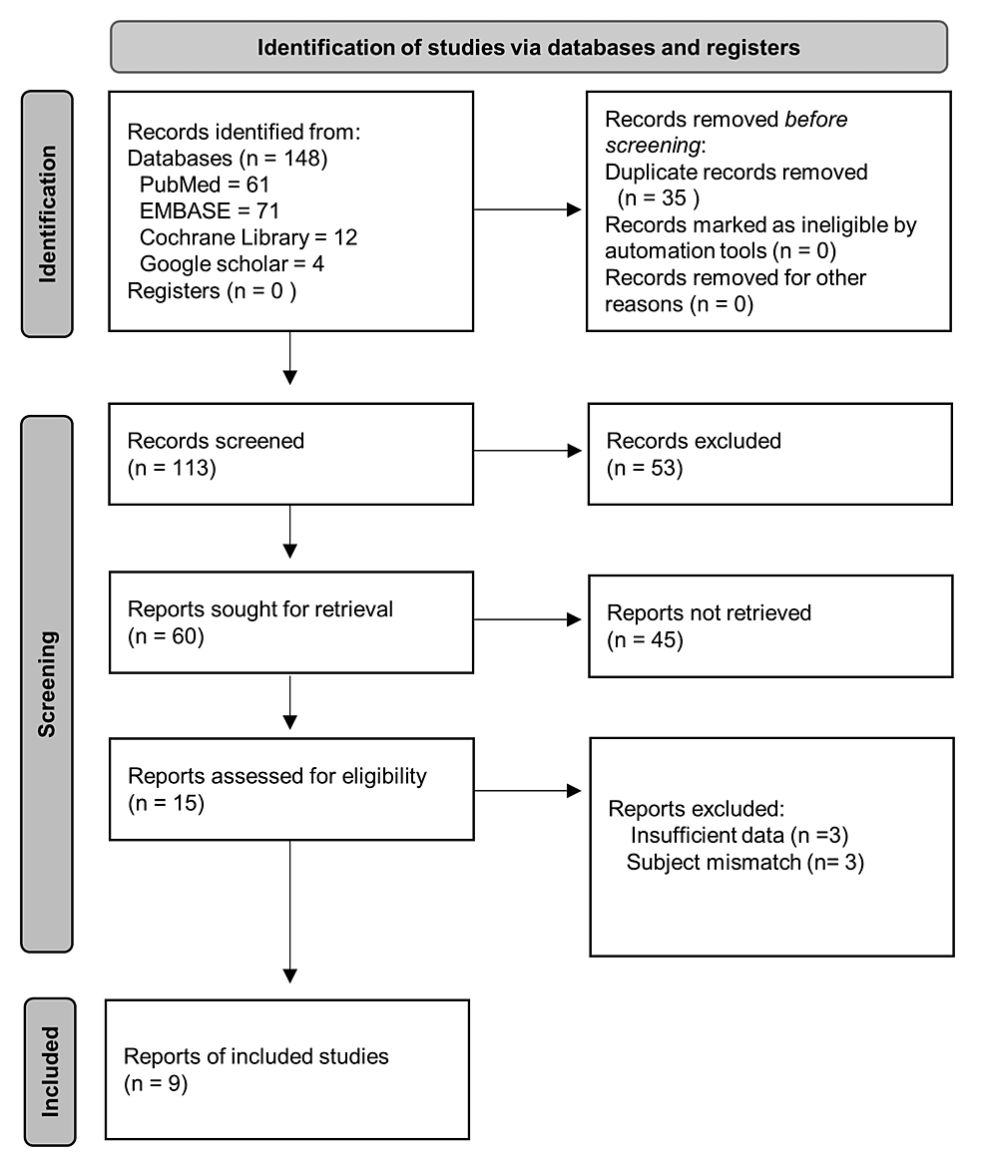
Flow diagram of the study process

Figure 1 shows a screening flowchart. A total of 148 studies were initially identified and published from 1985 to 2023. Of these, 35 were excluded due to duplication. The abstracts of the remaining 113 studies were reviewed, reducing the number of studies to 15. After a full-text review, six studies were excluded owing to insufficient data and subject mismatches. Finally, nine studies were included in the meta-analysis (Table 1) [8-16]. These nine studies included children with acute gastroenteritis (548 cases and 7,819 controls). The presentation year was from 2009 to 2022. All studies were retrospective, and the mean NOS score was 7.33 (Table 2).

**Table 1:** Characteristics of the included studies * Significant differences (p-values) were reanalyzed independently by us using a summarized t-test, citing the reference’s sample size and test values. § Conversion of units was performed regarding the data in the original paper. ¶ Means and standard deviations integrated regarding the data from the original paper. Abbreviations: UA, uric acid; SD, standard deviation; R, retrospective study

First author	Year	Country	Age range (month)	Sex (%male)	Sample size (exposure)	Sample size (control)	Serum UA levels (exposure) (mg/dL) (mean ± SD)	Serum UA levels (control) (mg/dL) (mean ± SD)	Significant difference ^*^	Study design
Motoyama M [16]	2009	Japan	9-33	61.46	13	83	-	-	-	R
Tsujita Y [8]	2011	Japan	20.36 ± 11.59 ^¶^	46.00	20	30	10.00 ± 2.20	8.80 ± 5.20	p = 0.268	R
Kang B [14]	2013	Korea	20.96 ± 14.71 ^¶^	56.95	59	696	-	-	-	R
Chae SH [9]	2014	Korea	16.41 ± 7.48 ^¶^	55.81	27	188	8.75 ± 2.31	5.91 ± 2.45	p < 0.001	R
Ma X [15]	2019	China	4-96	57.14	55	50	-	-	-	R
Yoo IH [10]	2019	Korea	8-38	54.81	154	2938	9.79 ± 2.16	6.04 ± 2.38	p < 0.001	R
Yoo SY [11]	2020	Korea	1-83	56.52	89	2701	8.20 ± 2.34	5.44 ± 2.69	p < 0.001	R
Fang C [12]	2022	China	1-60	52.57	82	93	8.03 ± 1.93 ^§^	5.06 ± 1.53 ^§^	p < 0.001	R
Jiang D [13]	2022	China	2-53	59.55	49	40	9.49 ± 2.10 ^§^	5.69 ± 2.37 ^§^	p < 0.001	R

**Table 2:** NOS for the included studies Abbreviation: NOS, Newcastle-Ottawa Scale

First author	Year	Selection	Comparability	Outcome/exposure	Total score
Motoyama M [16]	2009	****	**	**	8
Tsujita Y [8]	2011	****	**	**	8
Kang B [14]	2013	****	**	**	8
Chae SH [9]	2014	****	*	**	7
Ma X [15]	2019	****		**	6
Yoo IH [10]	2019	****		**	6
Yoo SY [11]	2020	****	*	**	7
Fang C [12]	2022	****	**	**	8
Jiang D [13]	2022	****	**	**	8

Meta-Analysis

**Figure 2:**
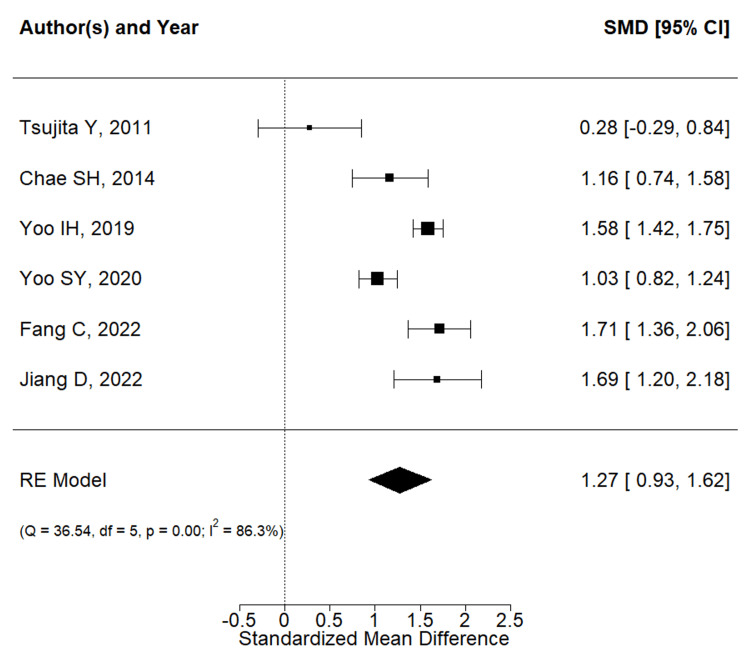
Forest plot of studies on CwG and serum UA levels Tsujita Y [8], Chae SH [9], Yoo IH [10], Yoo SY [11], Fang C [12], and Jiang D [13] Abbreviations: CwG, benign convulsions with mild gastroenteritis; UA, uric acid; SMD, standardized mean difference; CI, confidence interval, RE, random-effects

The meta-analysis showed that the laboratory finding most strongly associated with CwG was an increase in UA levels (SMD = 1.27, N = 6,411; 95% CI: (0.93, 1.62); Z = 7.258, p < 0.001; *I*^2^ = 86.32%, p < 0.001) (Table 3), followed by decreased Na level (SMD = -0.70, N = 1,468; 95% CI: (-1.02, -0.38); Z = -4.316, p < 0.001; *I*^2^ = 75.92%, p < 0.001), decreased chlorine (Cl) level (SMD = -0.61, N = 1,204; 95% CI: (-0.91, -0.31); Z = -3.989, p < 0.001; *I*^2^ = 57.44%, p = 0.052), and decreased WBC level (SMD = -0.53, N = 1,267; 95% CI: (-0.70, -0.36); Z = -6.217, p < 0.001; *I*^2^ = 0.00%, p = 0.428). Apart from this, minor significant differences were confirmed in the laboratory findings, including decreased hydrogen carbonate (HCO_3_^-^ ) level (SMD = -0.38, N = 3,231; 95% CI: (-0.52, -0.23); Z = -5.053, p < 0.001; *I*^2^ = 0.00%, p = 0.504), increased aspartate transaminase (AST) level (SMD = 0.32, N = 1,274; 95% CI: (0.15, 0.49); Z = 3.635, p < 0.001; *I*^2^ = 46.90%, p = 0.052), and decreased blood urea nitrogen (BUN) level (SMD = -0.31, N = 1,099; 95% CI: (-0.52, -0.10); Z = -2.859, p = 0.004;*I*^2^ = 0.00%, p = 0.887). No significant differences were observed in the C-reactive protein (CRP), potassium (K), Ca, glucose (Glu), alanine aminotransferase (ALT), and creatinine (Cre) levels. In particular, for UA, where the effect size was quantified as large (SMD > 0.8), a physiologically significant difference was considered to exist and subsequently analyzed (Figure 2). We identified a report by Tsujita et al. [8] as a single outlier. Repeating the meta-analysis without this outlier, we obtained a pooled estimate of 1.42 (N = 6,361; 95% CI: (1.12, 1.72); Z = 9.242, p < 0.001; *I*^2^ = 81.68%, p = 0.002), resulting in a percentage variation of approximately 5% in *I*^2^ (Table 3). High heterogeneity indicates unreliability but does not appear to be a severe inconsistency as the effect estimates are in the same direction.

**Table 3:** Summary of the effect size for all parameters * Calculated using the fixed-effects model owing to low heterogeneity. Abbreviations: SMD, standardized mean difference; CI, confidence interval; WBC, white blood cell; CRP, C-reactive protein; Na, sodium; K, potassium; Cl, chlorine; Ca, calcium; Glu, glucose; AST, aspartate transaminase; ALT, alanine aminotransferase; UA, uric acid; BUN, blood urea nitrogen; Cre, creatinine; HCO_3_^-^, hydrogen carbonate

			Effect size				Heterogeneity		
Parameter		Study (number)	Pooled SMD	95% CI	Z-value	p-value	*I*^2^ (%)	Tau-squared	p-value
WBC (10^3^/µL) *		5	-0.53	(-0.70, -0.36)	-6.217	<0.001	0.00%	-	0.428
CRP (mg/dL) *		3	-0.16	(-0.39, 0.07)	-1.324	0.186	6.43%	-	0.344
Na (mEq/l/L)		7	-0.70	(-1.02, -0.38)	-4.316	<0.001	75.92%	0.137	<0.001
K (mEq/L)		5	-0.15	(-0.56, 0.25)	-0.746	0.456	75.86%	0.156	0.002
Cl (mEq/L)		5	-0.61	(-0.91, -0.31)	-3.989	<0.001	57.44%	0.065	0.052
Ca (mg/dL)		5	-0.19	(-0.47, 0.10)	-1.295	0.195	60.02%	0.062	0.040
Glu (mg/dL)		4	-0.25	(-0.72, 0.23)	-1.006	0.315	81.00%	0.190	0.001
AST (IU/L) *		5	0.32	(0.15, 0.49)	3.635	<0.001	46.90%	-	0.110
ALT (IU/L) *		6	-0.03	(-0.19, 0.12)	-0.424	0.672	0.00%	-	0.990
UA (mg/dL)	Original	6	1.27	(0.93, 1.62)	7.258	<0.001	86.32%	0.148	<0.001
UA (mg/dL)	Outlier removed	5	1.42	(1.12, 1.72)	9.241	<0.001	81.68%	0.090	0.002
BUN (mg/dL) *		4	-0.31	(-0.52, -0.10)	-2.859	0.004	0.00%	-	0.887
Cre (mg/dL) *		4	-0.19	(-0.40, 0.02)	-1.743	0.081	28.84%	-	0.239
HCO_3_^-^ (mmol/L) *		3	-0.38	(-0.52, -0.23)	-5.053	<0.001	0.00%	-	0.504

**Figure 3:**
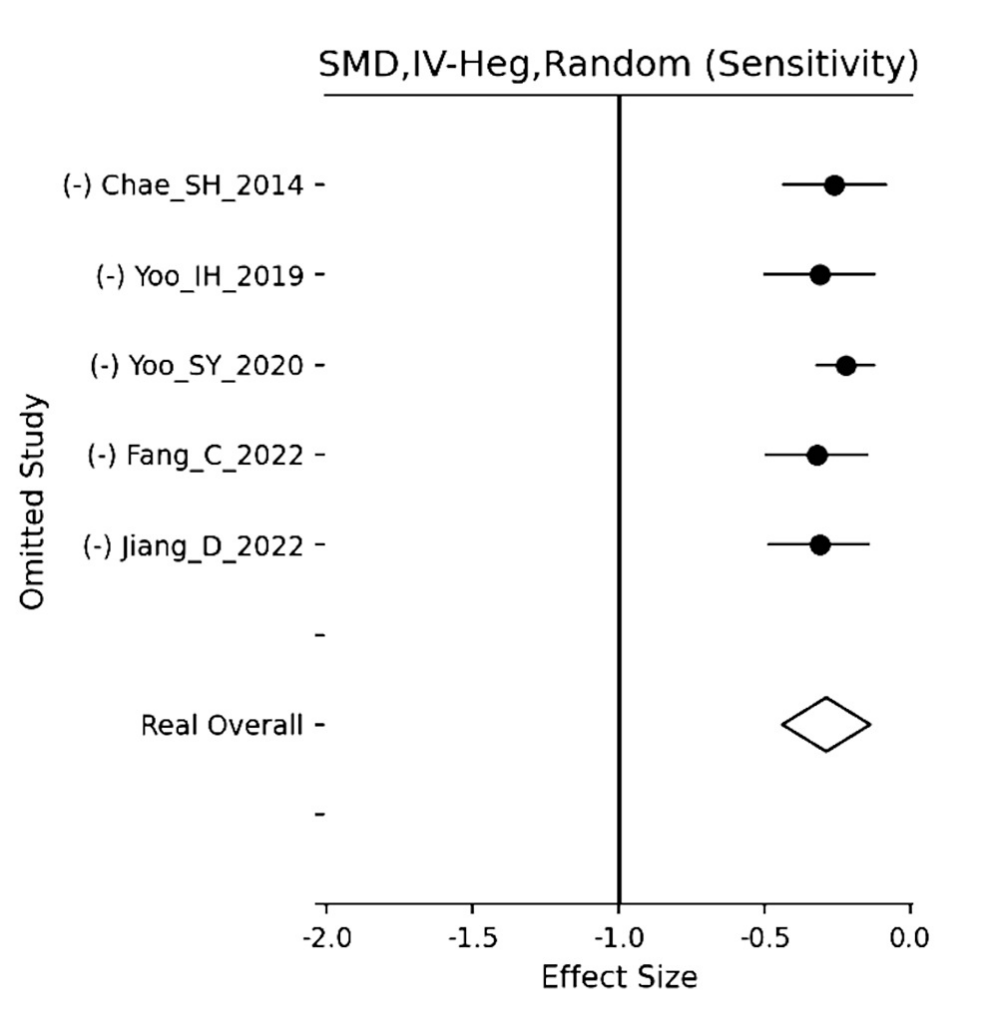
Sensitivity analysis of studies on CwG and serum UA levels without an outlier Chae SH [9], Yoo IH [10], Yoo SY [11], Fang C [12], and Jiang D [13] Abbreviations: CwG, benign convulsions with mild gastroenteritis; UA, uric acid, SMD, standardized mean difference

Sensitivity analysis after removing the outlier verified no significant change in the pooled SMD (Figure 3). Therefore, we concluded that there was a significant association between increased serum UA levels and CwG.

Meta-Regression and Subgroup Analysis

High heterogeneity indicated unreliability; therefore, we performed further meta-regression analysis for numerical variables and subgroup analysis for categorical variables. The results of the univariate meta-regression analysis between effect size and possible contributors to heterogeneity were insignificant (Table 4).

**Table 4:** Univariate meta-regression analysis between effect size and possible contributors to heterogeneity Abbreviations: WBC, white blood cell; Na, sodium; Ca, calcium

	Study (number)	Coefficients	p-value	*I*^2^	Tau-squared	R^2^
Publication year	5	1.018	0.384	72.65%	0.047	19.56%
Age	5	0.967	0.405	70.21%	0.044	24.40%
Sex	5	-0.465	0.674	71.75%	0.049	16.64%
WBC	3	-3.437	0.180	0.00%	-	100.00%
Na	3	-0.816	0.564	0.78%	0.001	98.58%
Ca	3	-4.792	0.131	0.00%	-	100.00%

**Figure 4:**
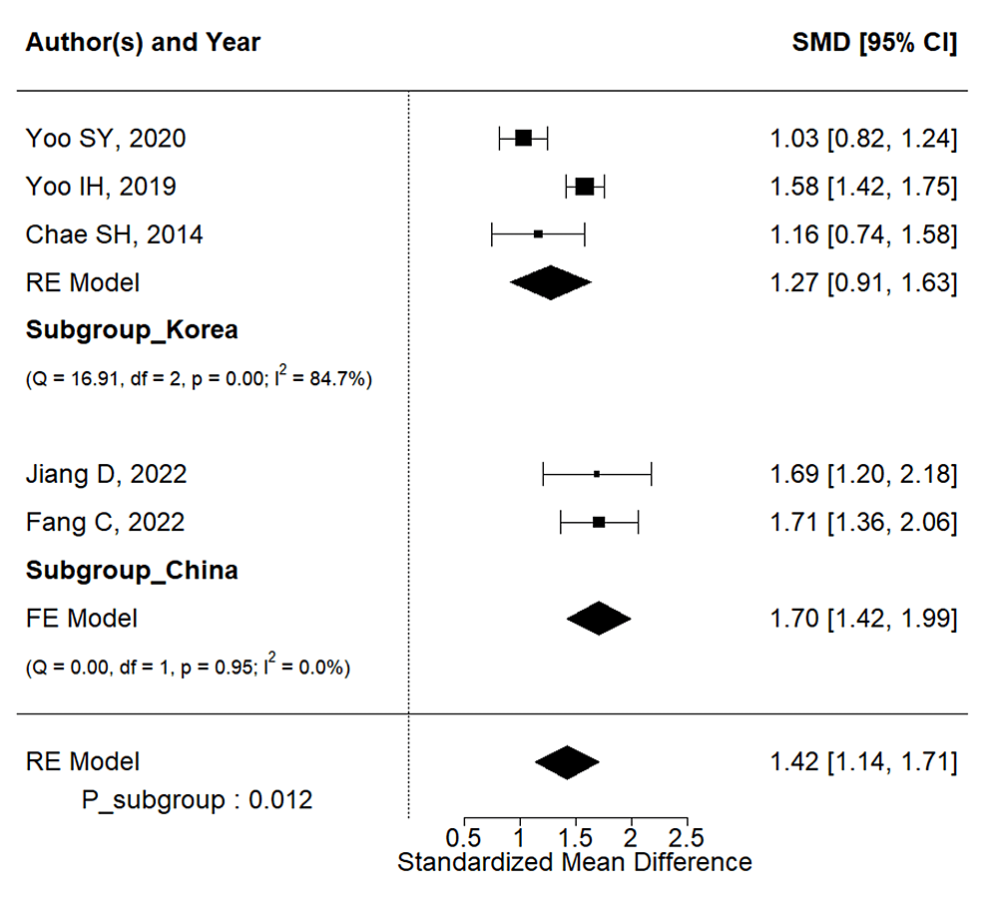
Result of subgroup analysis by country (Korea and China) Yoo SY [11], Yoo IH [10], Chae SH [9], Jiang D [13], and Fang C [12] Abbreviations: SMD, standardized mean difference; CI, confidence; RE, random-effects; FE: fixed-effects

For subgroup analysis, we classified the data into two subgroups by country. One was the Korean group, and the other was the Chinese group. We found heterogeneity between the Korean and Chinese subgroups (p = 0.012) (Figure 4), suggesting that the regional differences were one reason for this high heterogeneity.

Receiver Operating Characteristic Curve (ROC)

**Figure 5:**
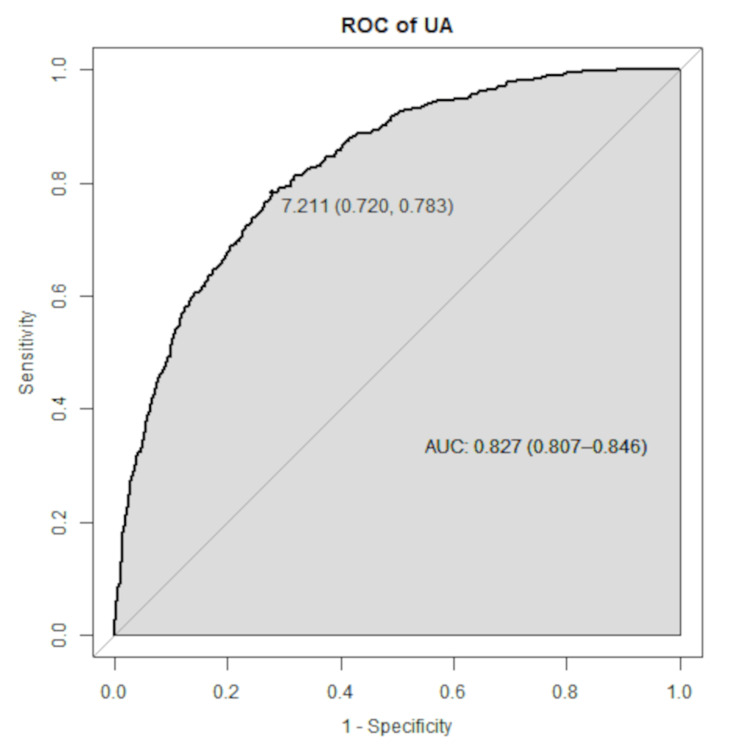
ROC for the prediction of CwG based on serum UA levels Abbreviations: ROC, receiver operating characteristic curve; CwG, benign convulsions with mild gastroenteritis; UA, uric acid; AUC, area under the receiver operating characteristic curve

Moreover, we searched for the thresholds of serum UA levels that caused CwG in five articles that excluded the outlier. As shown in Figure 5, the optimal cutoff value of the serum UA level was 7.21 mg/dL, with an area under the receiver operating characteristic curve (AUC) of 0.827 (95% CI: (0.807, 0.846)), a sensitivity of 72.0%, and a specificity of 78.3%. The performance of the classifier was moderate.

Discussion

Our study is the first meta-analysis to examine laboratory findings using CwG as an exposure group. In this meta-analysis, the laboratory findings associated with CwG included increased levels of UA and AST, and decreased levels of Na, Cl, WBC, HCO_3_^-^, and BUN. UA had the highest SMD among these laboratory findings. Therefore, focusing especially on UA as a predictor of the onset of convulsions in CwG, we concluded that a serum UA level could be a useful marker (optimal cutoff level: 7.21 mg/dL).

The association between abnormalities of blood test results and CwG is a controversial topic. Some studies have reported no blood test result abnormalities associated with CwG [5-7]. However, several other reports have documented increased UA levels [8-13], decreased Na levels [9,12,14,15], and decreased HCO_3_^-^ levels [13] in CwG compared to patients with mild gastroenteritis without convulsions. Fang et al. [12] reported that an elevated UA level was an independent risk factor for CwG, as per multifactorial logistic regression analysis. Moreover, Yoo et al. [11] reported significantly higher serum UA levels in CwG patients compared to those with acute gastroenteritis due to rotavirus and norovirus infections. They proposed that the optimal cutoff value of serum UA level to distinguish CwG from acute gastroenteritis was 7.35 mg/dL, with a sensitivity of 70.7%, a specificity of 77.7%, and an AUC of 0.789 (95% CI: (0.745-0.833)) [11]. Although the definition of serum standard UA levels in children and adolescents is unclear as these levels change during development [21,22], in adults, serum UA > 7.0 mg/dL is widely used to define hyperuricemia [23]. Hence, the reported cutoff values are considered high. Our results further support their findings that CwG is associated with hyperuricemia.

Hyperuricemia primarily results from underexcretion, which occurs via two primary pathways, the kidney (70%) and the intestine (30%) [24]. Dehydration with CwG can decrease UA excretion from the kidneys. However, in many patients with mild gastroenteritis, at least moderate to severe dehydration is unlikely to occur. Moreover, there is a report that UA values do not change when corrected for HCO_3_^-^ [10]. In our meta-analysis, we found a slight association between decreased BUN levels and CwG. BUN levels increase with dehydration, so the effect of dehydration on CwG in this study is unlikely. Although the possibility that UA levels increased due to dehydration caused by gastroenteritis cannot be ruled out, this is an issue for future studies.

Regarding the intestinal pathway, acute gastroenteritis can also cause hyperuricemia [23]. A study on conditions associated with hyperuricemia in over 9,000 hospitalized pediatric patients found that gastroenteritis was the most common cause [22]. Among the causative agents of gastroenteritis, rotavirus infection is most likely to cause hyperuricemia [25,26]. The UA excretion factor ATP-binding cassette transporter G2 (ABCG2), which inhibits UA excretion not only from the kidney but also from the intestine, may fail to function in gastroenteritis [27]. Matsuo et al. [28] proposed that one possible mechanism is that injury to the intestinal epithelium due to gastroenteritis decreases intestinal UA excretion via ABCG2.

This meta-analysis included papers from Japan, Korea, and China, primarily involving individuals of Asian descent [8-16]. The missense variant Q141K within ABCG2 is highly prevalent in some Asian subgroups [29], suggesting the possibility that genetic abnormalities may be involved in the increased UA levels observed in CwG due to impaired kidney and intestinal UA excretion pathways.

The relationship between seizures and UA suggests that UA may contribute to seizure susceptibility [30,31]. Research has shown that UA, with its pro-inflammatory and pro-oxidative properties, leads to increased excitability and severity of epileptic seizures [32]. Based on these findings, it is plausible that UA accumulation in the blood due to impaired UA excretion from the intestinal tract caused by acute gastroenteritis is involved in the development of CwG. However, further studies are necessary to confirm this hypothesis.

The strength of our study is that it was the first meta-analysis to investigate the relationship between CwG and UA levels. In addition, we used sensitivity analysis to evaluate the evidence and identify specific reference values to predict CwG. However, this study has some limitations. First, all studies included were retrospective. This may have introduced several biases that could have affected the results. Second, a high degree of heterogeneity was observed among the studies. Third, articles from non-indexed and unpublished journals were not included, which may have resulted in publication bias. Fourth, this study has not been able to completely rule out other factors that may cause elevated UA, such as dehydration.

## Conclusions

Our results suggest that the most prevalent laboratory finding was an increased serum UA level with a large effect, and the optimal serum UA cutoff value was 7.21 mg/dL with moderate accuracy. Our report strongly supports the known finding of elevated UA levels in CwGs. This meta-analysis also showed that increased serum UA levels were significantly associated with CwG. As for the causal role of hyperuricemia in CwG, larger prospective studies with exposure to hyperuricemia and outcomes of CwG are needed.

## Figures and Tables

**Figure 6:**
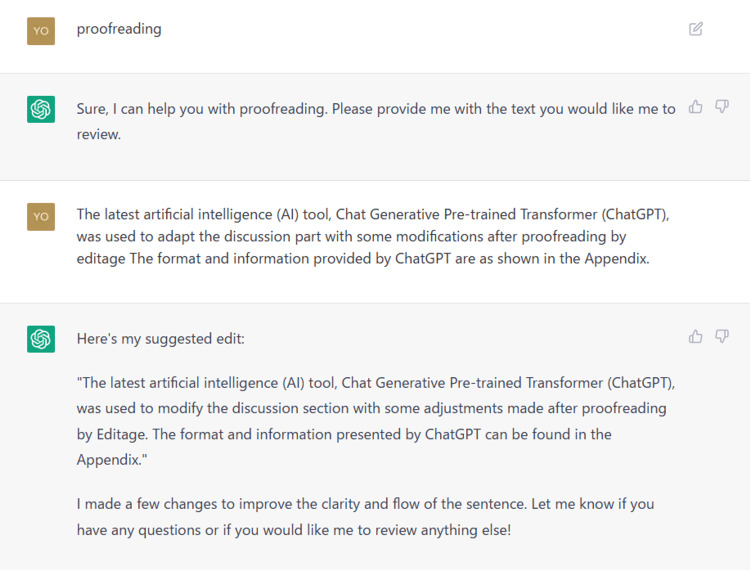
ChatGPT

**Figure 7:**
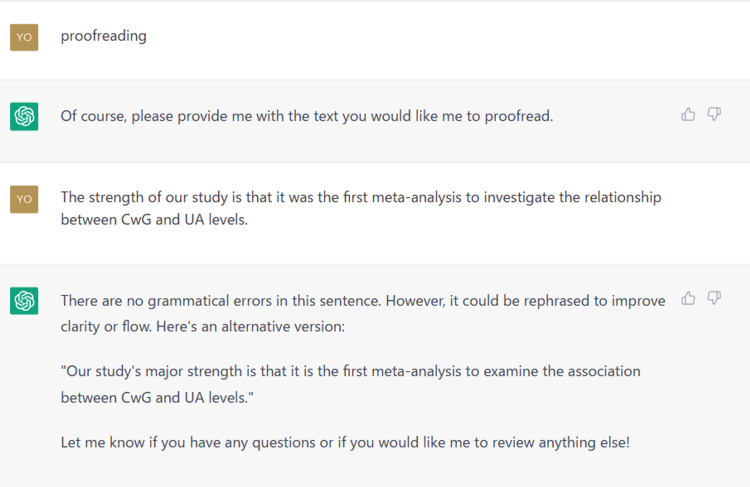
ChatGPT

## Appendices

The latest artificial intelligence (AI) tool, Chat Generative Pre-trained Transformer (ChatGPT), was used to modify the discussion section with some adjustments made after proofreading by Editage (https://www.editage.com/). The format and information presented by ChatGPT can be found in Figure 6 and Figure 7.
